# Data for health system comparison and assessment in the African Region: A review of 63 indicators available in international databases

**DOI:** 10.7189/jogh.14.04118

**Published:** 2024-06-21

**Authors:** Katherine Polin, Nathan Shuftan, Erin Webb, Daniel Opoku, Benson Droti, Wilm Quentin

**Affiliations:** 1 Department of Healthcare Management, Technische Universität Berlin, Berlin, Germany; 2 European Observatory on Health Systems and Policies, Brussels, Belgium; 3 German West-African Centre for Global Health and Pandemic Prevention, Kumasi, Ghana; 4 Kwame Nkrumah University of Science and Technology, Kumasi, Ghana; 5 Health Information Systems, World Health Organization Regional Office for Africa, Brazzaville, Congo; 6 Chair of Planetary & Public Health, University of Bayreuth, Bayreuth, Germany

## Abstract

**Background:**

Achieving universal health coverage in the African region requires health systems strengthening. Assessing and comparing health systems contributes to this process, but requires internationally comparable data. The European Observatory on Health Systems and Policies has produced Health Systems in Transition (HiT) reviews in Europe, Asia, North America and the Caribbean with a standardised template. This study explores data availability in international databases for the quantitative health and health system indicators in the HiT template for the WHO African region.

**Methods:**

We identified ten databases which contained data for 40 of the 80 original HiT indicators and an additional 23 proxy indicators to fill some gaps. We then assessed data availability for the resulting 63 indicators by country and time, i.e. first/last year of data, years of data available overall and since 2000, and we explored for each indicator (1) against the country with the greatest availability overall and (2) against annual availability for all years since 2000.

**Results:**

Overall data availability was greatest in South Africa (93.0% of possible total points) and least in South Sudan (59.5%). Since 2000, Uganda (60.4%) has had the highest data availability and South Sudan (37.2%) the lowest. By topic, data availability was the highest for health financing (91.4%; median start/end date 2000/2019) and background characteristics (88.5%; 1990/2020) and was considerably lower for health system performance (54.5%; 2000/2018) and physical and human resources (44.8%; 2004/2013). Data are available for different years in different countries, and at irregular intervals, complicating time series analysis. No data are available for service provision indicators.

**Conclusions:**

Gaps in data in international databases across time, countries, and topics undermine systematic health systems comparisons and assessments, regional health systems strengthening, and efforts to achieve universal health coverage. More efforts are needed to strengthen national data collection and management and integrate national data into international databases to support cross-country assessments, peer learning, and planning. In tandem, more research is needed to understand the specific historical, cultural, administrative, and technological determinants influencing country data availability, as well as the facilitators and barriers of data sharing between countries and international databases, and the potential of new technologies to increase timeliness of data.

Health systems strengthening (HSS) is high on global health research and policy agendas and is key to achieving universal health coverage (UHC) and Sustainable Development Goal (SDG) 3 [1,2]. It requires improving the performance of and relationship between single health system building blocks [3,4]. Health systems across the World Health Organization (WHO) African Region face variable, complex, persistent, and emerging challenges to HSS [5–7].

Systematic health system comparisons and assessments can support HSS [8,9]. They are useful for examining different approaches to health system organisation, financing, and service provision, and can highlight issues in system performance and help identify underlying causes, foster knowledge exchange, and inform policy and programme design [10]. Different methodological approaches and tools exist for these purposes [11–14], including the Health Systems in Transition (HiT) review template from the European Observatory on Health Systems and Policies (referred to as ‘Observatory’ from here onwards), which has helped produce reports on nearly 60 national health systems across Europe, North America, and the Asia-Pacific region [15,16].

Standardised, regularly updated, multi-country data on a common set of indicators is a prerequisite for systematic reviews of health systems [8,9]. Given the difficulty of accessing and the burden of processing and standardising national data for many countries, systematic cross-country comparisons and assessments could benefit from data available in international (global and regional) databases. While several studies have explored the availability of such internationally comparable data in the European region and high-income countries [17,18], similar research in the WHO African Region is scarce and often focussed on national level health information systems (HIS) [19,20].

Despite this gap, understanding the landscape of available international data for health and health system indicators in the WHO African region is essential, as many (if not all) countries in the region depend to some degree on external technical and/or financial support for health [21]. Moreover, development partners and international donors often rely on international databases to identify priorities and quantify results, so the lack of international data may negatively impact the alignment of international support with national needs.

In this study, we explore data availability in international databases for the 47 countries of the WHO African region (Appendix S1 in the **Online Supplementary Document**) for indicators of health system characteristics and performance included in the HiT template, as a recent review of templates found the indicators in the HiT to be among those commonly used for systematic health system analysis [22]. More specifically, we aim to identify relevant international databases and assess data availability for indicators by country, temporal breadth (first and last year of data reporting), and temporal depth (number of years with data per country).

## METHODS

### HiT template as benchmark

**Figure 1:**
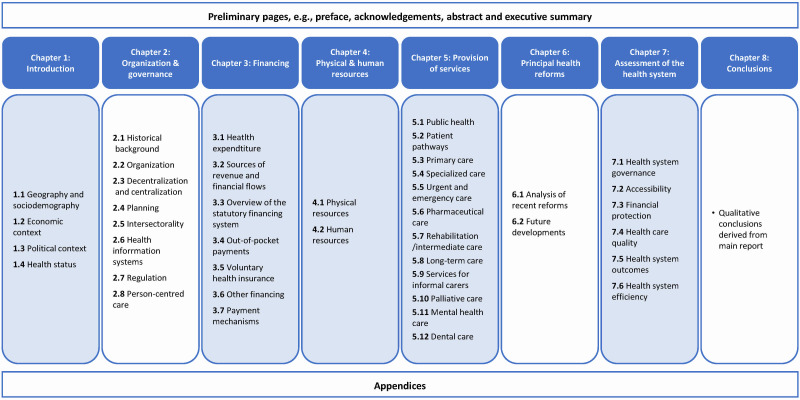
Structure of chapters and included topics of the HiT template. Shading indicates chapters with quantitative indicators which were assessed for data availability; chapters without shading ask for qualitative information which was excluded in this review. Source: adapted from [15].

One hundred and eighteen qualitative and quantitative indicators are included in the HiT template, organised into eight chapters corresponding to specific topics and sub-topics (**Figure 1**) [15]. While qualitative information provides important context, this review focusses on quantitative indicators for their comparability. After we excluded 38 qualitative indicators primarily related to organisation and governance or health reforms, 80 quantitative indicators remained for background characteristics (introduction) and features of health financing; physical and human resources; service provision; and health system performance assessment (HSPA).

### Data search and databases

We initially searched for data in 2020, but updated the search between November 2022 and February 2023. The HiT template refers to five global and four European or high-income country databases, the latter of which are not relevant for the WHO African Region. We thus searched for additional databases using Google, snowballing, and via discussions with regional experts and health system researchers. This led to the identification of five additional databases for a total of ten global or regional databases (**Table 1**).

**Table 1:** Databases used in this study (in alphabetical order)

Database name	Short description	Number of indicators covered	Website
**Recommended in the European Observatory’s HiT template**			
IHME, Global Health Data Exchange, Global Burden of Disease Database	Data catalogue providing information on population health from IHME, an independent global health research centre at the University of Washington.	8*	https://www.healthdata.org/gbd/2019
Transparency International’s Corruption Perceptions Index	Index ranking countries ‘by their perceived levels of public sector corruption, as determined by expert assessments and opinion surveys.’	1	https://www.transparency.org/en/cpi/2021/index/dom
World Bank, World Development Indicators Database	Collection of development indicators presenting the most current and accurate global development data available; includes national, regional, and global estimates.	29	https://databank.worldbank.org/reports.aspx?source=world-development-indicators
WHO, Global Health Expenditure Database	Database of internationally comparable data on health spending for close to 190 countries.	5*	https://apps.who.int/nha/database/
WHO, Global Health Observatory Database	Gateway to health-related statistics for WHO’s 194 Member States, including over 1000 indicators on mortality and burden of diseases, the MDGs, SDGs, NCDs and risk factors, epidemic-prone diseases, environmental health, violence and injuries, health systems and human resources, and equity.	10	https://www.who.int/data/gho/data/themes/topics
**Additional data sources**			
Afrobarometer’s Merged Data Database	Regular public attitude surveys on democracy, governance, the economy and society in >30 countries.	1	https://www.afrobarometer.org/data/merged-data/
IMF, DataMapper Database	Large collection of IMF data sets, including regional and country economic indicators.	2	https://www.imf.org/external/datamapper/profile/OEMDC/WEO
Primary Healthcare Performance Initiative	Partnership convening policymakers, health system managers, advocates and other development partners to catalyse improvements in primary health care in LICs and LMICs through better measurement and knowledge-sharing.	2	https://improvingphc.org/measuring-primary-health-care-performance
UNICEF Data Warehouse	Collection of databases of hundreds of international valid and comparable indicators across many countries.	1	https://data.unicef.org/dv_index/
WHO Integrated African Health Observatory	Tool to monitor achievements in health systems strengthening in the region. Data are derived from WHO databases as well as national sources.	5	https://aho.afro.who.int/data-and-statistics/af

IHME – Institute for Health Metrics and Evaluations, IMF – International Monetary Fund, LIC – low-income countries, LMICs – low- and middle-income countries, MDG – Millenium Development Goals, NCD – non-communicable disease, SDG – Sustainable Development Goal, UNICEF – United Nations Children’s Fund, WHO – World Health Organization.

*Denotes that this source is also used in one composite indicator.

### Selection of indicators

**Figure 2:**
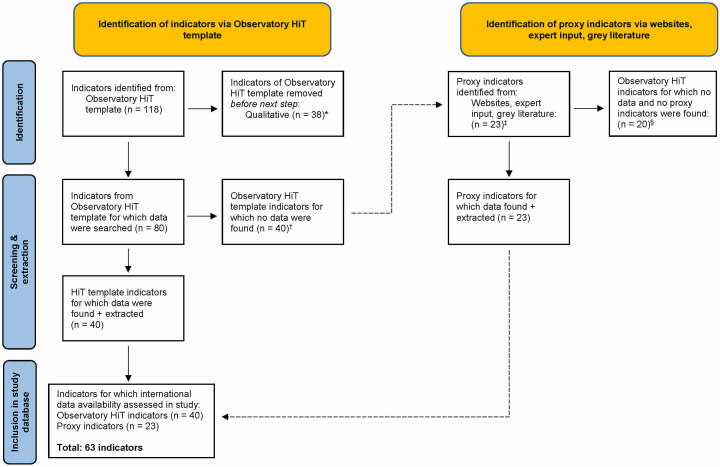
Flowchart depicting indicator inclusion for data availability assessment. *Mainly in health system organisation and governance (chapter 1); provision of services (chapter 5); and principal health reforms (chapter 6). †From background (chapter 1); financing (chapter 3); physical and human resources (chapter 4); provision of services (chapter 5); and assessment of the health system (chapter 7). ‡For missing indicators in background (chapter 1); financing (chapter 3); physical and human resources (chapter 4); and provision of services (chapter 5) and assessment of the health system (chapter 7). §We found multiple proxy indicators for some indicators, and none for others.

**Figure 3:**
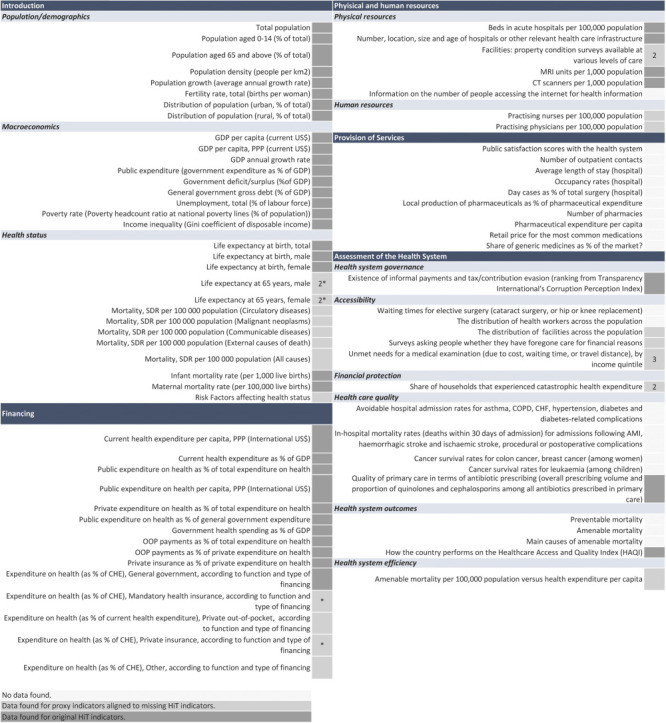
Specific indicators for which any data were available by type of indicator (e.g. original or proxy). AMI – acute myocardial infarction, CHE – current health expenditure, CHF – congestive health failure, COPD – chronic obstructive pulmonary disorder. *Same proxy indicator used, numerical values (e.g. 1, 2, 3) refer to the number of proxy indicators found for given original HiT indicators.

Of the 80 quantitative HiT indicators, 40 were unavailable in the identified databases. However, we explored thematically related proxy indicators for which data existed in the ten different databases. In this way, we identified 23 proxy indicators providing metrics on conceptually similar indicators to those with missing data [23] (**Figure 2**). For example, the original HiT indicator ‘Life expectancy at 65 years, male’ was unavailable in the assessed databases, but we identified ‘Life expectancy at 60 years, male’ as a proxy indicator. Similarly, the original HiT indicators for health expenditure data disaggregated by financing schemes and functions were unavailable, but we identified ‘Total health care expenditure by health care functions (USD millions)’ as a proxy indicator (**Figure 3**; Appendix S2 in the **Online Supplementary Document**). Meanwhile, for 20 HiT indicators, we could find neither data nor proxy indicators.

Ultimately, we assessed data availability for 63 indicators. Data for the same indicators were sometimes available from multiple sources. In such cases, we assessed the availability of data in the database with the most data available across countries and time. As we aimed to check the availability of data across indicators and countries, we did not analyse the data specifically (e.g. the reported values) and therefore did not check the consistency of available data, the plausibility of the results, or data quality, including breaks in series. Capturing data availability, however, may serve as a valuable diagnostic tool and impulse for further research into these areas.

### Analysis

We assessed the availability of data for indicators across countries and time and captured it in a matrix in Microsoft Excel (Office 365 version; Microsoft Corporation, Redmond, Washington, USA), in which we also performed all statistical analyses. We investigated two temporal dimensions: breadth (first year to last year) and depth (total years available within breadth and since 2000) (Appendix S2 in the **Online Supplementary Document**). As there is no benchmark or ‘gold standard’ for total data availability, we defined a measure for relative data availability by assigning the largest temporal depth observed (i.e. the value of the country with the highest number of data years available) as each indicator’s denominator.

We calculated indicator availability for a particular country by dividing the respective temporal depth by said denominator. For example, with 12 years of data available for ‘Beds in acute hospitals per 100 000 population’, Benin had the largest observed temporal depth for that indicator, due to which we defined 12 years as both the denominator for this indicator and as 100% relative data availability. We also summed indicator denominators to calculate a hypothetical maximum number of total data points by topic and overall. To assess overall relative data availability per country, we added country temporal depth values across all indicators and divided each country’s total temporal depth by this aggregate. Given the importance of having recent data for policy decisions, we separately examined absolute data availability since 2000. As we felt normatively that most countries in the WHO African region should have regular data available across indicators since 2000, we defined 23 years (2000–22) as 100% data availability.

## RESULTS

### Databases

Among the ten global and regional databases identified and included in this review, the World Bank’s World Development Indicators Database was the most dominant, followed by the World Health Organization’s Global Health Observatory, covering 29 and ten indicators across several topics, respectively, and reflecting the broadest temporal breadth and depth among data sources (**Table 1**; Appendix S2 in the **Online Supplementary Document**). The Institute for Health Metrics and Evaluation covering eight indicators is the third most prominent database. The metadata showed that data are frequently from the same national sources, from purposeful expert surveys or modelled estimates.

### Available indicators

There were 63 indicators (n = 40 HiT; 23 proxy) in the ten international databases for which any data were available, while 20 HiT indicators had neither data nor proxy indicators (**Figure 3**). Of the 63 indicators with any data: 31 indicators related to background characteristics (n = 22 HiT; 9 proxy); 14 on health financing (n = 11; 3); 8 on physical and human resources (n = 4 HiT; 4 proxy); and 10 on HSPA (n = 3 HiT; 7 proxy). No data are available, and no proxies were found for indicators related to service provision (n = 10), health care quality (n = 4), outcomes (n = 3), and accessibility (n = 2), and physical resources (n = 1).

### Overall relative data availability per country

**Figure 4:**
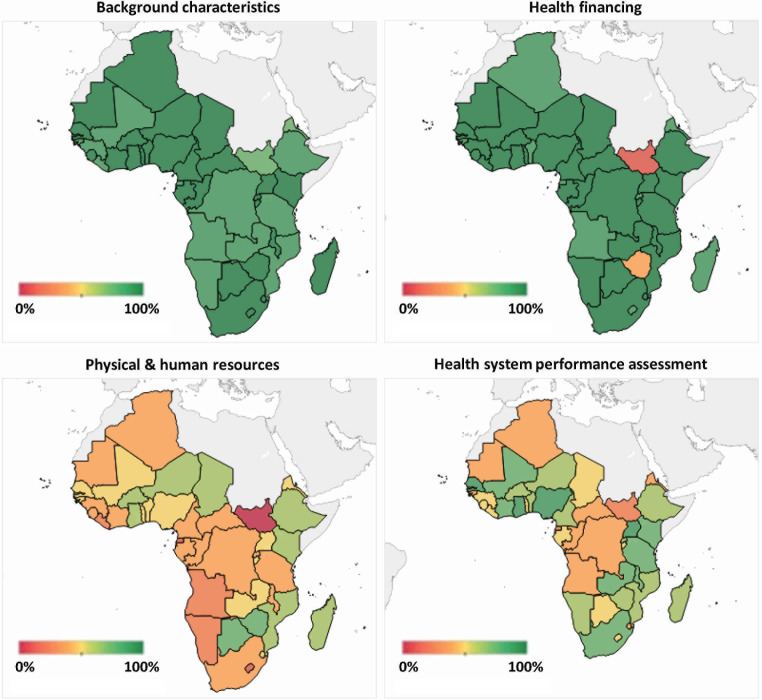
Overall relative data availability by country and topic. The colour scales are diverging. Green refers to greater availability per country, yellow to medium availability and red to low availability. We calculated country data availabilities by dividing respective summated temporal depths by summated denominators per topic.

For indicators where data were available in at least one country, the maximum, aggregate data availability across all indicators and years was 1739 possible data points (‘100% data availability’) (**Figure 4**). South Africa has the highest relative data availability over time with 1618 data points (93.0%), while South Sudan has the lowest with 1035 (59.5%). Almost all countries have high data availability for background characteristics (average data availability 88.5% of 1375 total possible data points, standard deviation (SD) = 72.1); and all but South Sudan and Zimbabwe have data available for most health financing indicators (91.4% of 203 total possible data points, SD = 25.6). There are gaps in data on physical and human resources everywhere (44.8%; of 66 total possible data points, SD = 9.4) and for several HSPA-related indicators (54.5% of 95 total possible data points, SD = 13.9).

Looking at overall relative data availability by the geographical subregions of the WHO African Region (Western, Central, Eastern, Southern), the averages are similar for background characteristics and health financing (heavily influenced by less data availability in Eritrea and South Sudan) (**Table 2**). For physical and human resources, Southern Africa has the highest subregional average, though also the highest SD (due to low availability in Lesotho and Namibia), followed by Eastern Africa (high SD due to Comoros and South Sudan). Finally, Southern Africa has the highest average overall availability for HSPA-related indicators.

**Table 2:** Subregional averages and SDs for overall relative data availability, with SDs in brackets*

Chapter	Topic	Western Africa	Central Africa	Eastern Africa	Southern Africa
1	Background characteristics	89.9% (46.8)	88.4% (59.9)	85.5% (104.8)	89.7% (49.1)
3	Health financing	94.6% (7.1)	93.0% (6.0)	87.1% (43.4)	89.0% (28.2)
4	Physical and human resources	46.5% (5.5)	37.0% (7.7)	47.1% (11.4)	47.3% (11.8)
7	Health system performance	57.6% (14.1)	43.6% (7.9)	54.6% (16.5)	60.7% (8.1)

*Western Africa includes Algeria, Benin, Burkina Faso, Cabo Verde, Cote d'Ivoire, Gambia, Ghana, Guinea, Guinea Bissau, Liberia, Mali, Mauritania, Niger, Nigeria, Senegal, Sierra Leone, and Togo. Central Africa includes Angola, Burundi, Cameroon, Central African Republic, Chad, Congo (Republic of), Democratic Republic of Congo, Equatorial Guinea, Gabon, and Sao Tome and Principe. Eastern Africa includes Comoros, Eritrea, Ethiopia, Kenya, Madagascar, Mauritius, Rwanda, Seychelles, South Sudan, Uganda, and United Republic of Tanzania. Southern Africa includes Botswana, Eswatini, Lesotho, Malawi, Mozambique, Namibia, South Africa, Zambia, and Zimbabwe. Source: adapted from [24].

### Data availability since 2000

**Figure 5:**
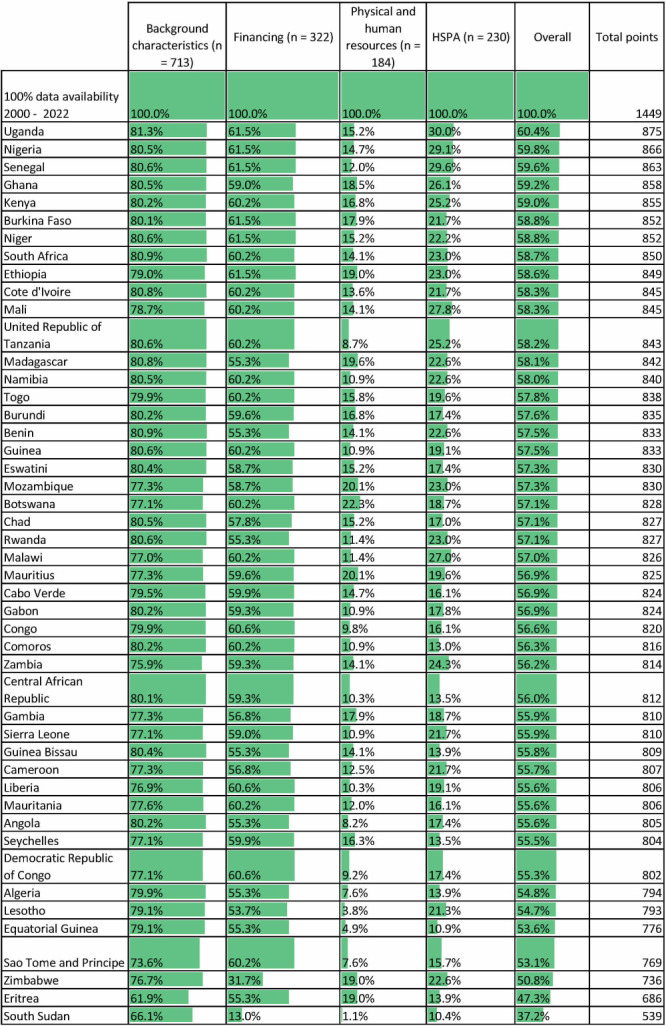
Data availability across countries since 2000. N represents the total number of data points, calculated as the total number of indicators in a topic × 23 years × 47 countries.

Given our normative decision that countries should have regular data available across indicators since 2000, we defined maximum data availability (‘100% data’) as 1449 data points (23 years × 63 indicators) (**Figure 5**). Average data availability was 815.5 data points (56.2%, SD = 54.1), which is lower than overall data availability (85.3%, SD = 97.3) given the different benchmarks. Uganda has the most points (n = 875, 60.4%), followed by Nigeria (n = 866, 59.8%) and Senegal (n = 863, 59.6%). Eritrea (n = 686, 47.3%) and South Sudan (n = 539, 37.2%) have the least. Mirroring overall trends, data availability was best for background characteristics (average data availability 78.5% of 713 total possible data points, SD = 25.2) and health financing (57.4% of 322 total possible data points, SD = 25.5), and lowest for physical and human resources (13.4% of 184 total possible data points, SD = 8.3) and HSPA (20.1% of 230 total possible data points, SD = 11.2).

Concerning subregional averages and SDs for absolute data availability across countries since 2000 (**Table 3**), the largest SDs across topics can be seen in the Eastern Africa subregion. This is likely due to low data availability in South Sudan, but also in Eritrea, Tanzania, Comoros, Rwanda, and Seychelles for specific topics.

**Table 3:** Subregional averages and SDs for absolute data availability across countries since 2000, with SDs in brackets*

Chapter	Topic	Western Africa	Central Africa	Eastern Africa	Southern Africa
1	Background characteristics	79.5% (9.9)	78.8% (14.9)	76.8% (44.8)	78.3% (12.8)
3	Health financing	59.3% (7.0)	58.5% (6.3)	54.7% (43.1)	55.9% (28.3)
4	Physical and human resources	13.8% (5.2)	10.5% (6.2)	14.4% (10.3)	14.6% (9.7)
7	Health system performance	21.1% (10.9)	16.5% (6.2)	20.0% (13.9)	22.2% (6.2)

*Western Africa includes Algeria, Benin, Burkina Faso, Cabo Verde, Cote d'Ivoire, Gambia, Ghana, Guinea, Guinea Bissau, Liberia, Mali, Mauritania, Niger, Nigeria, Senegal, Sierra Leone, and Togo. Central Africa includes Angola, Burundi, Cameroon, Central African Republic, Chad, Congo (Republic of), Democratic Republic of Congo, Equatorial Guinea, Gabon, and Sao Tome and Principe. Eastern Africa includes Comoros, Eritrea, Ethiopia, Kenya, Madagascar, Mauritius, Rwanda, Seychelles, South Sudan, Uganda, and United Republic of Tanzania. Southern Africa includes Botswana, Eswatini, Lesotho, Malawi, Mozambique, Namibia, South Africa, Zambia, and Zimbabwe. Source: adapted from [24].

### Data availability over time

**Figure 6:**
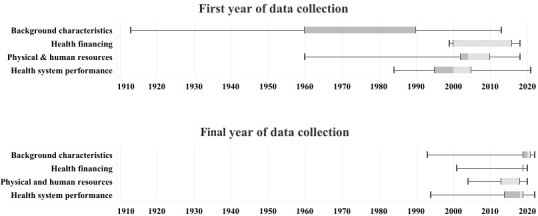
Temporal breadth of data availability by topic.

Data on background characteristics have been available for several countries since the 1960s. For most indicators and countries, they have been available from 1990 until relatively recent periods (median last year: 2020) (**Figure 6**). For health financing, data have been available for most indicators and countries from 2000 until relatively recently (median last year: 2019); however, some indicators (e.g. ‘Out-of-pocket payments as a % of private expenditure on health’ have only been available by 2012. For physical and human resources, data became available for most countries only after 2000 (median first year: 2004), and recent data are unavailable for most indicators (median last year: 2013). Data for some HSPA-related indicators have been available since 1984, but most have been available only since 2000 (median first year) and until 2018 (median last year).

Data on background characteristics have the highest temporal depth on average (39.3 years), which is just below their temporal breadth (41.5 years on average), indicating that data are available for almost every year in each country since the start of data collection, in part due to modelled estimates (**Table 4**). Similarly, for health financing, average temporal depth is almost identical to temporal breadth, indicating that data have been available for most years since 2000. Data on physical and human resources have been missing for many years in most countries, as reflected by average temporal depth (3.7 years) and breadth (10.4 years). Data for HSPA-related indicators have also been regularly reported, with average temporal depth (5.2 years) being several years below temporal breadth (13.4 years). **Table 5** shows the subregional averages for the temporal breadth and temporal depth of data available.

**Table 4:** Temporal depth of data availability by topic

Chapter	Topic	Average temporal breadth (in years)	Average temporal depth (in years)	Countries with no data available for at least one indicator
1	Background characteristics	41.5	39.3	18
3	Health financing	12.8	13.3	10
4	Physical and human resources	10.4	3.7	47 (all)
7	Health system performance	13.4	5.2	39

**Table 5:** Subregional averages for the temporal breadth and temporal depth of data available*

Temporal breadth (in years)					
**Chapter**	**Topic**	**Western Africa**	**Central Africa**	**Eastern Africa**	**Southern Africa**
1	Background characteristics	42.2	41.3	40.1	42.3
3	Health financing	13.3	13.2	12.2	12.3
4	Physical and human resources	10.8	10.9	9.1	10.4
7	Health system performance	14.4	10.9	12.8	15.3
**Temporal depth (in years)**					
**Chapter**	**Topic**	**Western Africa**	**Central Africa**	**Eastern Africa**	**Southern Africa**
1	Background characteristics	39.9	39.2	37.9	39.8
3	Health financing	13.7	13.5	12.6	12.9
4	Physical and human resources	3.8	3.1	3.9	3.9
7	Health system performance	5.5	4.1	5.2	5.8

*Western Africa includes Algeria, Benin, Burkina Faso, Cabo Verde, Cote d'Ivoire, Gambia, Ghana, Guinea, Guinea Bissau, Liberia, Mali, Mauritania, Niger, Nigeria, Senegal, Sierra Leone, and Togo. Central Africa includes Angola, Burundi, Cameroon, Central African Republic, Chad, Congo (Republic of), Democratic Republic of Congo, Equatorial Guinea, Gabon, and Sao Tome and Principe. Eastern Africa includes Comoros, Eritrea, Ethiopia, Kenya, Madagascar, Mauritius, Rwanda, Seychelles, South Sudan, Uganda, and United Republic of Tanzania. Southern Africa includes Botswana, Eswatini, Lesotho, Malawi, Mozambique, Namibia, South Africa, Zambia, and Zimbabwe. Source: adapted from [24].

## DISCUSSION

Systematic health system comparisons and assessments can support HSS, but require the availability of standardised, regularly updated, multi-country data on a common set of indicators. Ours is the first study to have systematically assessed data availability in international databases for a standard set of 80 indicators for health system comparisons and assessments for the 47 countries in the WHO African region.

Previous studies have focussed on national data collection and management capacity, as well as indicator coverage from household surveys in order to understand health and health system-related data availability in the African region [19]. However, indicators in international databases are essential for cross-country comparative analyses and are often used by international organisations or donors to identify priority areas for national support. Conducting international health system assessments based on data that are available only at the national level or buried within surveys would not be feasible. Therefore, similar to previous studies conducted on data availability in Europe [25], our study focusses on the availability of data in international databases.

In line with other studies exploring the availability of data across a range of health and health systems indicators [26], we found that data for the African region was dispersed across several sources. We identified ten global or regional databases that provide data for the indicators we assessed in this study. Of the original 80 indicators, only 40 have any data available at all. Twenty-three proxy indicators with data available can fill some of these gaps. By geography, South Africa has the most data available over time, while South Sudan has the least. Uganda and Nigeria have the most data available since 2000.

By topic, almost all countries have high data availability for background and health financing indicators (except South Sudan and Zimbabwe). Indeed, by time and geography, data on background characteristics have been available annually for most countries for almost four decades, while data on health financing have been available annually for most countries, but only since 2000. Recent data (after 2019/2020) for the indicators surveyed in both of these topics is unavailable. Meanwhile, there are large gaps in data for indicators on physical and human resources and several HSPA-related indicators. Timewise, indicators for HSPA and physical and human resources have been available only for certain countries, were often not reported regularly, and are mostly out-of-date. No data are available for health service provision.

Our study identifies patterns in data availability overall and by topic, e.g. where data are more frequently available for background and health status/risk indicators than for health systems and services. These findings are similar to those found for the European region. For the 88 European Core Health Indicators (ECHI) [25], highest data availability was identified for aspects of demography and socio-economic situation (about 98 · 0% of 9 indicators), followed by health status (just over 90% of 25 indicators) and determinants of health (about 88.0% of 12 indicators). Indicators pertaining to health services had the lowest availability of data (about 87.5% of 23 indicators). The one indicator on health promotion had 100.0% availability of data for the countries assessed. Similar data availability was also found in a study assessing the availability of health and health services data for the WHO global reference list of 100 core health indicators for Sierra Leone alone [26,27]. Using primarily international and some national level data sources, including Demographic and Health Surveys, the availability of data for a much smaller selection of indicators in Sierra Leone ranged (from highest to lowest) from 95.0% for risk factors to 88.0% for health status, down to 63.0% for health services and 25.0% for health systems indicators. Notably, this study also incorporated proxy indicators for one-fifth of its indicators [26].

Though our study found that international databases lack any data on service provision indicators and have very limited data on physical and human resources, countries in the region do routinely collect health facility data on service provision, supplies, and human resources [19]. Therefore, the missing data in international databases suggests a disconnect between the national and international levels. Reasons for this may include issues with completeness, timeliness, and quality of national data, as well as communication challenges in the relationship with international organisations [19]. When data are not reported by national authorities to international databases, these organisations often have to rely on modelled estimates to fill gaps [28]. However, these estimates do not necessarily reflect conditions on the ground and may overrepresent the availability of national-level data for certain indicators. Using these estimates may lead to distortions in priority setting and programme design of international organisations and donors.

Our results show other important data gaps, including in timeliness (only about half of possible data points have been available since 2000) and large geographic variation in overall relative data availability across the entire region (93.0% for South Africa vs 59.5% in South Sudan (**Figure 4**)) and within subregions (**Table 2**, **Table 3**). Previously, the UHC monitoring report provided an overview of data gaps and variation across countries [29], focussing mostly on indicators measured from Demographic and Health Surveys and Multiple Indicator Cluster Surveys. It found similar geographic variation in data availability. Existing data disparities and the lack of recent data, especially for physical and human resources and HSPA, raise questions about the usefulness of this data for HSS efforts. Without recent data, researchers and policy advisors are bound to rely on outdated information, hampering efforts to strengthen evidence-informed policymaking [30]. Countries with notably large gaps in data availability in international databases (as identified in our study) will require particular attention and support to improve their national data collection and reporting capacities [19].

Our finding that long-term data availability in international databases has been best for background characteristics and relatively good for health financing since 2000 likely reflects the priorities of international donors and international organisations. During the 1960s, the United Nations (UN) system focussed on macroeconomic targets related to demographic growth and economic development, while global attention on maternal, neonatal, and child health and sanitation standards was growing in the 1970s [31]. This is mirrored by the first years of data availability of related indicators. Health financing has meanwhile received increasing attention from the late 1990s to the early 2000s, which is again reflected by the first years of data collection of related indicators. Nevertheless, several health financing indicators are still unavailable; for example, data on health spending by health care function and type of insurance is rarely available. Additionally, recent health financing data (after 2020) is still unavailable.

The countries in the region are diverse in terms of colonial history (e.g. whether a Belgian, British, Dutch, or French colony), time of independence, socialist history, country administrative structure (e.g. whether a federal or centralized system), recent history of civil war and conflict, and economic status and growth as represented by changes in gross domestic product (GDP). While there does not seem to be a correlation between many of these characteristics, except possibly recent history of civil war and conflict, with divergent levels of data availability, an in-depth quantitative analysis of the relevance of these factors would need to be addressed in a different paper.

This study has several limitations. First, the indicators surveyed derive from the Observatory’s HiT template. While most indicators are relevant for the WHO African region, some may only be relevant for certain countries (e.g. those with established insurance schemes or with more mature health information management systems) [32]. Yet other indicators relevant for the African region might be missing; for example, the HiT template does not include indicators for mortality from malaria or HIV/AIDS or unmet need for family planning services, which are important HSPA indicators in the region. Currently, the African Health Observatory Platform on Health Systems and Policies [33] is developing and testing a template for the region’s country health system and services profiles, while the WHO Regional Office for Africa is developing its African Core Health Indicators. The latter also uses the 100 core health indicators as a benchmark reference [27]. Future research will need to assess the availability and policy relevance of data for these projects. Second, and in relation to this, we found that half of the original HiT indicators do not have any data available, especially for health service provision. This calls into question, as we mentioned above, the relevance of these indicators for the region. This also may suggest that total data availability was worse than we found in the study, as we used the number of indicators for which any data were found (and excluded half the HiT indicators for which there were no data) as the denominator, rather than the entire set of HiT indicators. Third, we focussed exclusively on global and regional databases. Data found to be unavailable may be available nationally, e.g. service provision data, but were not reported internationally or could be extracted from household and facility-based surveys. Conversely, data available internationally may not be available nationally because they are, in fact, generated by international organisations’ computing estimates via modelling and interpolation to fill data gaps. Therefore, our results should not be misinterpreted as providing an overview of data availability at the national level. Fourth, our study conducted numerous searches using Google, snowballing and discussions with regional experts and health system researchers. Yet, it is possible that a database was missed, which could potentially have provided complementary information. Lastly, the intent of our study was to assess the availability of data for specific health and health system indicators as informed by the Observatory’s HiT template. As such, we did not specifically check the data itself, including consistency across databases or issues of quality. We also did not check the plausibility of the results beyond locating and contextualizing them in the current literature. Understanding the accessibility, quality, and consistency of the data available, among other things, are important future research priorities and are essential to strengthening data capacity in the region.

Nevertheless, this research has important implications for policymakers and researchers. First, the almost complete absence of recent data on many important aspects related to HSS, e.g. physical and human resources, service provision and aspects of HSPA, limit the potential of systematic analysis of health systems based on internationally available data to foster knowledge exchange or inform policy decisions in the WHO African region. Gaps in data for these indicators hamper evidence-based HSS policy efforts and are a missed opportunity for advancing UHC and improving health system performance and, most importantly, population health. Greater efforts are needed to assure recent information on these topics is available in international databases. National data sources may include information on resources as well as service provision, yet unlike for high-income countries, this information is rarely available in international databases for African countries. International organisations may need to support national governments to prioritise supplying data to international databases, including with technical and/or financial support. Supporting capacity building nationally would enable the region’s countries to have more control of the health- and health system-story narrative and reduce reliance on international organisation-generated estimates, which can lack accuracy. As countries in the WHO African region often rely on development aid for health and HSS programming, improved access to data could also lead to more targeted development programming based on accurate data-driven insights.

Second, to enhance HSS, data on health systems and performance must be easily accessible for researchers and policymakers. Currently, data are scattered across ten main global and regional databases and myriad smaller data sources or surveys, complicating comparative cross-country research. There is a case for a unified health system performance data platform for the African region as a whole, or at least for individual countries in the region [26]. Such a platform could be, for example, a single data repository or a data partnership, ensuring that what data exists nationally is reported internationally and standardised. This could involve a formal, equitable collaboration [34] with governments, researchers, survey funders and managers as well as development partners and other organisations that generate or manage relevant data, with initiatives like the integrated African Health Observatory [35] and African Health Observatory Platform on Health Systems and Policies serving as inspiration or being leveraged and built on to improve data gathering and analysis.

Third, our findings underline the need for future research in several important areas. An investigation of the cultural and historical determinants of data collection and management may provide useful insights and signposting. Further, apart from assessing data availability on a more contextualised set of indicators, the quality and consistency of data available in and across international databases should be explored. It is also important to better understand the availability of data on service provision and HSPA in national sources and explore the potential of standardising these for reporting internationally. As national data collection, management, and reporting shapes the availability of data at the international level, further exploration of the capacity, resources, and bottlenecks in countries with consistently lower data availability (e.g. South Sudan and Eritrea) and for specific topics as identified in the subregional analysis since 2000 is essential, given how important recent data are for HSS. Future research should also attempt to identify and understand the facilitators and barriers to data sharing between the national and international levels (e.g. health system development, HIS maturity, and health research landscape).

Finally, real-time data generation and monitoring are becoming widespread [36] through advances in and the proliferation of new technologies. In particular, the COVID-19 pandemic catalysed significant innovation in health and health care data generation, collection, and exchange worldwide [37]. It is therefore increasingly difficult to justify the lack of recent data in international databases on key areas for HSS in the African region, as this undermines constructive, evidence-based health policymaking and opportunities to improve population health. More research is needed to explore the challenges and potential solutions of new technologies to facilitate the reporting of up-to-date information on common health system indicators to international databases.

## CONCLUSIONS

The availability of standardised, regularly updated data on health system indicators in international databases can support cross-country analyses and assessments, foster peer learning, and contribute to improved planning and programming. We found that data on health systems is scattered across ten international databases; data on existing indicators have important gaps, as information is often missing for several countries and years, particularly in view of service provision, physical resources, and health system accessibility, quality, and outcomes; and recent data (after 2020) are very rarely available for any indicators.

The implications of these findings are threefold. First, greater efforts are needed to strengthen national data collection and to ensure reporting of existing national data to international databases; these efforts should focus on those countries with the greatest data gaps as identified in our study. Second, the integration of existing data into one central data repository or coordinated platform could facilitate data accessibility for cross-country analyses and peer-learning. Third, more research is needed to better understand the root causes and determinants of identified data gaps; to explore facilitators and barriers for data sharing between the national and international levels; to assess possibilities for standardising existing national data and the quality of data at the national and international levels; and to explore the potential of new technologies, including challenges and solutions, to increase timeliness and comprehensiveness of data. Progress is imperative across all three to close data gaps, as existing data disparities undermine targeted and constructive evidence-based policymaking and HSS with negative impacts on population health.

## Additional material


Online Supplementary Document
